# Protective Effects of *Ligularia fischeri* and *Aronia melanocarpa* Extracts on Alcoholic Liver Disease (*In Vitro* and *In Vivo* Study)

**DOI:** 10.1155/2020/9720387

**Published:** 2020-04-14

**Authors:** Chang-Won Pyun, Tae-Su Seo, Dae-Jung Kim, Tae-Woo Kim, Jung-Shik Bae

**Affiliations:** ^1^R&D Laboratory, Wellfine Co., Ltd, 2-6, 32, Soyanggang-ro, Chuncheon-si, Gangwon-do, Republic of Korea; ^2^Well-being Bioproducts R&D Center, Kangwon National University, Gangwon 25209, 11, IT Valley-gil, Gonggeun-myeon, Hoengseong-gun, Gangwon-do, Republic of Korea

## Abstract

Hepatic protective effects of *Ligularia fischeri* (LF) and *Aronia melanocarpa* (AM) against alcohol were investigated *in vitro* and *in vivo* test. LF, AM, and those composed mixing material (LF+AM) were treated in HepG2 cell. Alcohol dehydrogenase (ADH) and acetaldehyde dehydrogenase (ALDH) activities were significantly increased in each singleness extract and mixed composite. The protective effect on alcoholic liver damage was investigated by animal models. Serum alcohol level and acetaldehyde level were significantly decreased by LF+AM treatment in acute experimental model. In the chronic mouse model study, we had found that the increased plasma liver damage index (alkaline phosphatase) by alcohol treatment was declined by oral administration of LF+AM extraction composite. As well as, it was identified that the protection effect was induced by increasing catalase activity and suppressing COX-2, TNF-*α*, MCP-1, and IL-6 mRNA expressions. CYP2E1 mRNA expression was also increased. These results suggest that oral ingestion of LF and AM mixed composite is able to protect liver against alcohol-induced injury by increasing alcohol metabolism activity and antioxidant system along with decreasing inflammatory responses.

## 1. Introduction

Alcohol consumption has become popular worldwide, particularly for relieving stress and for socializing [1, 2]. However, hangover after drinking may cause symptoms such as headache, vomiting, nausea, and muscle ache, while also causing mental and social detriment [3]. Moreover, excessive drinking may cause ischemic heart disease, pancreatic cancer, esophageal cancer, oral cancer, and diabetes [4]; moreover, it can be the cause of various liver diseases, including liver cirrhosis, hepatitis, and fatty liver [5]. Most habitual drinkers have alcoholic fatty liver, while 10-35% have alcoholic hepatitis, and 10-20% have alcoholic liver cirrhosis. Consequently, the seriousness of alcoholic liver diseases has become a major issue. In addition, the disease treatment is very costly, and thus, there is a growing trend in consumer demand for materials and foods, which can improve liver functions to prevent such diseases.

Alcohol absorbed into the body moves through the stomach and small intestines and is subsequently metabolized in the liver. Approximately 90% of alcohol absorbed in the liver is primarily converted into acetaldehyde by alcohol dehydrogenase (ADH), which is then secondarily oxidized into acetic acid by aldehyde dehydrogenase (ALDH). These metabolites of alcohol are ultimately degraded into water and CO_2_ for excretion through various routes, such as urine or sweat [6]. As indicated, metabolism of alcohol is regulated by the activities of ADH and ALDH, but when excessive amounts of alcohol are absorbed into the body, the metabolites accumulate inside the body due to the limited metabolic rate of ADH and ALDH. Acetaldehyde produced during alcohol metabolism is the major cause of toxic action that is more severe than the alcohol itself [7]. Acetaldehyde is a highly reactive compound, which interferes with mitochondrial respiration [8] and produces protein adducts, which are involved in hepatotoxicity [9, 10]. Here, the adducts produced bind directly with tubulin present in hepatocyte to impair material absorption and protein egress, which can induce immune responses that lead to liver damage [11]. In addition, significant amounts of reactive oxygen species produced during alcohol metabolism trigger lipid peroxidation, while the oxides cause damage to blood cells and cell membrane [12, 13] and produce reactive aldehydes to facilitate inflammation and liver fibrosis [14].

*Ligularia fischeri* (LF) is a perennial plant belonging to the *Asteraceae* family, which can grow up to 1 m tall, usually in wetlands deep in mountains. Its leaf is heart-shaped, and one stem usually has three leaves, growing up to 85 cm. Young leaves can be eaten raw as wraps or after slightly steaming and seasoning them in the Republic of Korea. LF consists of vitamins A, B_1_, B_2_, and C, as well as *β*-carotene and niacin. Among these, its vitamin A and *β*-carotene contents are known to be higher than in other vegetables [15]. It has generally been used as a therapeutic herb for bruises, low back pain, sputum, and coughs, while it has also been reported to have antioxidative [16], anticancer, anti-inflammatory [17, 18], and nitric oxide inhibition [19] effects. LF contains several iso forms of chlorogenic acid component such as 4-caffeoylquinic acid (4-CQA), 3,4-dicaffeoylquinic acid (3,4-dCQA), and 3,5-dicaffeoylquinic acid (3,5-dCQA) etc. Those phenolic compounds are reported as an antioxidative, antibacterial, and anti-inflammatory material [20].

*Aronia melanocarpa* (AM) is the name that collectively refers to a shrub belonging to the Aronia family of Rosaceae family in the order Rosales and its berries. It is an ornamental plant, which is also grown for its edible berries. *Aronia* can thrive in harsh environments, including subzero cold and strong ultraviolet ray environments. Reported reference materials include polymeric proanthocyanidins, chlorogenic acid, neochlorogenic acid, cyanidin-3-*O*-galactoside, and cyanidin-3-*O*-arabinoside [21], while it has been reported to have various physiological activities, including cardioprotective, antioxidant, anticancer, antimutagenic, and hepatoprotective activities [22]. Anthocyanins, which are major physiologically active substances, are the most abundant in AM among berries and widely used as functional materials in various foods and dietary supplements.

However, it has not been reported that the effect of both natural products (LF and AM) on alcohol induced liver injury. Therefore, to investigate the effects of extracts prepared from LF and AM on the alcoholic liver disease, the present study conducted experiments under *in vitro* and *in vivo* systems.

## 2. Materials and Methods

### 2.1. Preparation of Experimental Materials

Dried LF powder was purchased from an agricultural company Chorok Sarang (Jeongseon, Korea), and AM powder was purchased from local farm (Goseong-gun, Gangwon-do, Korea). LF extract was prepared by mixing LF powder with purified water at a ratio of 1 : 11 (w/w) and stirring for 60 min at 60°C. AM extract was prepared by mixing dried AM powder and 30% ethanol at a ratio of 1 : 13 (w/w) and stirring for 66 min at 25°C. The extracts obtained were vacuum filtered using Advantec filter paper No. 2 (CA, USA), after which, the filtrate was vacuum concentrated. The concentrated sample was dissolved in distilled water and freeze-dried for 72 h to prepare the LF and AM extracts, which were used for the further experiments. The yield of each freeze-dried extract powders for dried raw materials was 10.1% and 39.8%, respectively, in a lab scale process. As well as, to investigate the synergistic effect of LF+AM composite on alcoholic liver disease, each extract was evenly mixed at a ratio from 1 : 9 to 9 : 1 (w/w) for use in the experiment and had compared with each singleness extract material.

### 2.2. HPLC-RP Analysis

LF was dispersed in 50% EtOH (10 mg/mL) by vortex mixer. Dispersed solutions were sonicated for 10 min and centrifuged. The supernatant was used for HPLC analysis. AM was dissolved in 70% EtOH (5 mg/mL) and directly used for HPLC analysis. All samples were filtered using 0.45 *μ*m nylon syringe filter before injecting it into the HPLC system.

Chromatographic separations of caffeoylquinic acids (CQAs) were achieved using a Phenomenex Luna C18 column (250 mm × 4.6 mm I.D, 100 Å). A reverse phase HPLC was carried out by gradient conditions, and the flow rate of mobile phase was 1.0 mL/min. Mobile phases were composed in 0.1% triflouroacetic acid (TFA) in deionized water (A) and methanol (B). The gradient program started in 15% B and with the following composition: 0–3 min, 15% B; 3–7 min, 19% B; 7–15 min, 19% B; 15–28 min, 32% B; 28–33 min, 90% B; 33–38, 15% B; and 38–43, 15% B. Column oven was set at 30°C, and the detection of CQAs was performed at 330 nm on a UV detector.

C3G was determined by separating with a Phenomenex Luna C18 column (150 mm × 4.6 mm, 100 Å), and the mobile phases were composed of 0.4% of TFA in deionized water and 0.4% of TFA in 50% MeOH. The gradient program begun in 0% B: 0–2 min, 70%; 20–22 min, 100%, 22–30 min, 0% B; 30–35 min, 0% B. Temperature of separation was set at 30°C, and the detection wavelength was 520 nm.

### 2.3. HepG2 Cell Culture

Human hepatoma (HepG2) cells were obtained from the Korean Cell Line Bank (Seoul, Korea). HepG2 cells were cultured in Dulbecco's Modified Eagle Medium (DMEM) containing 10% heat-inactivated fetal bovine serum (FBS) and 1% penicillin-streptomycin under 5% CO_2_ condition with temperature maintained at 37°C.

### 2.4. Cell Viability Assay

Cell viability was measured using the MTT (3-[4,5-dime-thylthiazol-2-yl]-2,5-diphenyl-tetrazolium bromide) reduction method as previously described by [23]. After dispensing 100 *μ*L each of HepG2 cells (1 × 10^6^ cells/mL) into a 96-well plate and incubating it for 24 h, samples with concentrations of 0.05, 0.125, 0.25, and 0.5 mg/mL were prepared in medium without FBS and 1% penicillin-streptomycin. After incubating the treated cells with the samples at 37°C in 5% CO_2_ incubator for 24 h, 5 mg/mL of MTT solution, equivalent to 1/10 of DMEM, was added for 4 h and incubated at 37°C for MTT reduction. The medium was then removed while making sure the formazan generated did not escape with the medium. Subsequently, the cells were dried for 30 min in the dark, and 100 *μ*L of DMSO was dispensed and mixed for 1 h; after which, the absorbance was measured at 570 nm.

### 2.5. Animal Experiment 1: Effect of LF and AM on Alcohol Metabolism

Specific-pathogen-free (SPF) male Sprague Dawley (SD) rats (5-week-old) were purchased from DooYeol Biotech (Seoul, Republic of Korea). After 1 week of acclimation, following quarantine and inspection, healthy animals without any weight loss were selected for use in the experiment. The experimental animals were reared in an environment with the following settings: temperature of 23 ± 3°C, relative humidity of 50 ± 10%, ventilation cycle of 10–15 times/h, illumination time of 12 h (08 : 00–20 : 00), and illumination of 150–300 Lux. During the entire experimental period, the experimental animals were fed *ad libitum* with solid feed (Cargill Agri Purina, Seongnam, Republic of Korea) and drinking water.

LF-AM extract mixture (LF+AM) was used as the test article. After a 1-week acclimation period, healthy animals were selected and divided into six groups by randomized block design: normal control group (G1), alcohol-treated group (G2), alcohol +100 mg/kg body weight (BW) LF+AM-treated group (G3), alcohol +250 mg/kg BW LF+AM-treated group (G4), and alcohol +500 mg/kg BW LF+AM-treated group (G5). After the group assignment, the animals were fasted for 18 h. After fasting, the respective test article was administered orally, followed by oral administration of alcohol 30 min later. The test articles were dissolved in saline solution for oral administration, and the alcohol administered was fermented ethanol diluted to 40% at a concentration of 3 g/kg BW.

At 1 and 3 h after alcohol administration, orbital blood samples were collected. At 5 h after alcohol administration, the animals were anesthetized using isoflurane (VSpharm, Korea), and blood samples were collected from the heart. The collected blood samples were placed in separate serum tubes (Becton Dickinson, NJ, USA) and left at room temperature for 30 min. The tubes were centrifuged at 3,000 g for 10 min to separate the serum. The liver was extracted from animals that had completed the blood collection process, and the liver was washed with cold saline solution. The liver samples, along with the blood samples, were stored at -70°C until being analyzed. Animal experiment 1 was conducted according to the protocols approved by the Institutional Animal Care and Use Committee of Hallym University (approval number: Hallym 2018-49).

### 2.6. Animal Experiment 2: Protective Effects of LF and AM on Alcoholic Liver Damage

Specific-pathogen-free 5-week-old male ICR mice were purchased from DooYeol Biotech (Seoul, Republic of Korea) and used. After 1 week of quarantine, inspection, and acclimation process, healthy animals without weight loss were selected and used for the experiments. Experimental animals were reared in a feeding environment set at temperature of 23 ± 3°C, relative humidity of 50 ± 10%, ventilation frequency of 10–15 times/h, illumination time of 12 h (08 : 00–20 : 00), and illuminance exitance of 150–300 Lux. During the entire experimental period, experimental animals were fed *ad libitum* with solid feed for laboratory animals (Cargill Agri Purina, Seongnam, Republic of Korea) and drinking water.

After a 1-week acclimation period, healthy animals were selected and divided into five groups by randomized block design: normal control group (G1), alcohol control group (G2), alcohol +250 mg/kg body weight (BW) LF+AM-treated group (G3), and alcohol +500 mg/kg BW LF+AM-treated group (G4). Ten animals were used for each group, and the test substance was dissolved in drinking water and orally administered at a specific time every day for 4 weeks. The test substance was dissolved in drinking water and orally administered at a specific time every day for 4 weeks, and for alcohol treatment, 50% ethanol was orally administered at a dose of 10 mL/kg BW 1 h after administering the test substance. Animal experiment 2 was conducted according to the protocols approved by the Institutional Animal Care and Use Committee of Hallym University (approval number: Hallym 2018-76).

### 2.7. Serum Ethanol and Acetaldehyde Concentrations

Serum ethanol (Abcam, Cambridge, UK) and acetaldehyde (Megazyme, Bray, UK) were measured according to the manufacturer's protocol.

### 2.8. Measurement of Serum Liver Damage Indices and Lipid Components

To measure serum liver damage indices, the activities of alanine aminotransferase (ALT), aspartate aminotransferase (AST), alkaline phosphatase (ALP), gamma-glutamyl transpeptidase (*γ*-GT), etc. were measured. To confirm the extent of alcohol-induced abnormality in the body's lipid metabolism, the levels of serum triglyceride, total cholesterol, LDL-cholesterol, and HDL-cholesterol were measured by using a blood biochemistry analyzer (Konelab 20, Thermo Fisher Scientific, Massachusetts, USA).

### 2.9. Histomorphological Observation of the Liver

Liver tissue fixed in 4% paraformaldehyde (PFA) was embedded in paraffin, and 5 *μ*m tissue sections were produced from the embedded tissues. After removing paraffin, the tissues were hydrated by serially reducing the percentage of alcohol starting from 100% alcohol to 0% ethanol (H_2_O). For histomorphological observation of the liver tissue, the tissue was stained using an Accustain® Hematoxylin and Eosin Stains (Sigma-Aldrich Co.) according to the manufacturer's instruction, and then the histological changes in the liver tissue were observed using an optical microscope (Carl Zeiss).

### 2.10. Preparation of Liver Tissue Solution

Liver tissue solution was prepared for the measurement of alcohol metabolism-related enzymatic activities in the liver tissue. After adding 1 mL of PBS to 100 mg of liver tissue and homogenizing the mixture, the homogenate was centrifuged at 5,000 rpm for 10 minutes; after which, the supernatant was obtained for use as the liver tissue solution. Protein content in the liver tissue solution was measured using a BCA protein assay kit (Thermo Fisher Scientific, USA).

### 2.11. ADH and ALDH Activities

ADH and ALDH activities in HepG2 cells and liver tissue solution were measured according to the protocol given by the kit manufacturer (BioVision, CA, USA).

### 2.12. Measurement of Antioxidant Enzyme Activity and Lipid Peroxidation Level in Liver Tissue

To measure the activities of superoxide dismutase (SOD), catalase (CAT), and glutathione peroxidase (GPx) in the liver tissue, the respective measurement kits (Cayman Chemical) were used according to the manufacturer's instruction. Lipid peroxidation levels were measured by using a TBARS (thiobarbituric acid reactive substances) assay kit (Cayman Chemical) according to the manufacturer's instructions.

### 2.13. Investigation of Liver Tissue mRNA Expression (Real-Time RT-PCR)

Total RNA was isolated by using a TRIzol Reagent (Thermo Fisher Scientific) on liver tissue collected at the end of the experiment. Next, cDNA was obtained from total RNA (2 *μ*g) by using a HyperScriptTM RT master mix kit (GeneAll biotechnology); the mRNA levels of COX-2, iNOS, TNF-*α*, IL-6, MCP-1, and CYP2E1 genes were investigated by performing real-time PCR. The information on the primers used is shown in Table 1. For gene quantitative analysis, a Rotor-Gene 6,000 series system software 1.7 program (Corbett Research) was used.

### 2.14. Lipid Analysis

Lipids in the liver tissue were extracted by modifying the method previously described. Lipids were extracted by applying a chloroform : methanol (2 : 1 v/v) solution mixed to a fixed amount of liver tissue, agitated, and homogenized with a homogenizer. The mixture was centrifuged at 5,000 rpm for 10 min. The lipid-containing lower solutions were transferred to new tubes, and then pellets were obtained by evaporating chloroform via centrifugation for 2 h using a high efficiency centrifugal concentrator (Genevac). The lipids were later dissolved by applying chloroform to these pellets and then measured by using a triglyceride and total cholesterol quantitative measurement kit (Asan Pharm. Co., Ltd.) according to the manufacturer's instructions.

### 2.15. Statistical Analysis

All data were presented as mean ± SEM. The measurements were statistically processed using the one-way analysis of variance (ANOVA) test with Minitab® 19.2 (Minitab Inc., State College, PA, USA). The significance between each group and dose-dependent effect of LF+AM was performed by the Dunnett's test, and multiple comparison effect of all groups was performed by the Tukey's test.

## 3. Results and Discussions

### 3.1. Total CQA and C3G Content of LF and AM

**Figure 1:**
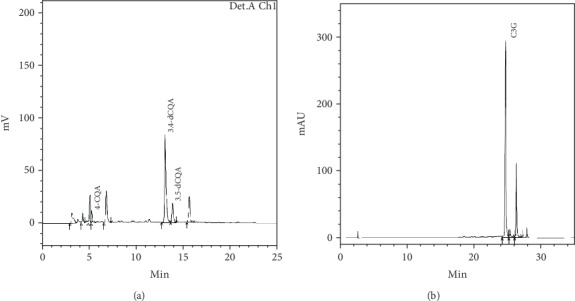
Typical chromatogram of *Ligularia fischeri* extract (a) and *Aronia melanocarpa* extract (b).

The typical chromatograms of LF extract and AM extract are expressed on Figure 1. Total CQA content of LF and C3G content of AM are 55.2 ± 2.19 mg/g and 27.0 ± 0.84 mg/g, respectively. These results are similar or lightly higher than those reported in previous studies [24, 25].

### 3.2. Effect of LF-AM Extract Complex on Cell Viability of HepG2

**Figure 2:**
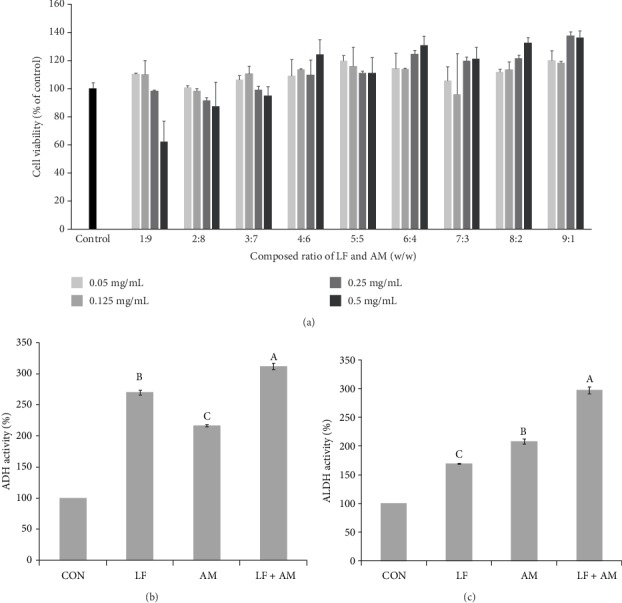
Cell viability of HepG2 cells according to the composition ratio of LF and AM (a), Effect of LF, AM, and LF+AM on ADH (b), ALDH (c) activity in HepG2 cells. ^a-d^Mean ± SD in the same row with different superscripts are significantly different at *p* < 0.05 CON, nontreated control; LF = *Ligularia fischeri* extract; AM = *Aronia Melanocarpa* extract; LF+AM = *Ligularia fischeri* extract and *Aronia Melanocarpa* extract mixed composite (9 : 1, w/w).

The results of the cytotoxicity of LF+AM on HepG2 cell line measured using MTT assay are shown in Figure 2(a). Relative to the control G1, cell viability of ≥80% was found in all samples with LF:AM composition ratio of 1 : 9 (w/w), except the sample with concentration of 500 *μ*g/mL.

### 3.3. Alcohol Dehydrogenase (ADH) and Aldehyde Dehydrogenase (ALDH) Activities in HepG2

The results of ADH and ALDH activities measured using HepG2 cell line according to the composition ratios of LF+AM are shown in Figures 2(b) and 2(c). Relative to the untreated group, the results were 270.1% for LF, 216.7% for AM, and 312.0% for LF+AM (9 : 1, w/w). As well as, the ALDH activities with LF, AM, and LF+AM (9 : 1, w/w) were 169.64%, 208.54%, and 297.2%, respectively. The LF+AM had shown higher ADH and ALDH activities than those of single material. These results suggest that LF+AM has synergistic effect on ADH and ALDH activities in HepG2 cell.

### 3.4. Animal Experiment 1: Effect of LF and AM on Alcohol Metabolism

#### 3.4.1. Blood Ethanol and Acetaldehyde Concentrations

In the group treated with alcohol and sterile purified water instead of the test article (negative control: G2), serum ethanol concentration at 1 h after alcohol administration was 0.83 ± 0.06 mM, which gradually decreased to 0.43 ± 0.03 and 0.30 ± 0.0 mM after 3 and 5 h, respectively. At 1 h after alcohol administration, there were no significant differences in serum ethanol concentrations based on the test articles. At 3 h after alcohol administration, groups treated with different concentrations of LF+AM (G3, G4, and G5) showed a trend of appreciable decrease in serum ethanol concentration, corresponding to increase in treatment concentrations. At 5 h after alcohol administration, 500 mg/kg BW LF+AM-treated group (G5) showed significantly lower serum ethanol concentration than the alcohol-treated group (G2), whereas the other experimental groups (G3 and G4) did not showed any difference relative to the alcohol-treated group (G2). The area under the curve (AUC), which represents the overall relationship of serum ethanol concentration over time, showed significant decrease based on the administration of LF+AM, with the largest decrease found in the group treated with high concentration of LF+AM (G5).

**Figure 3:**
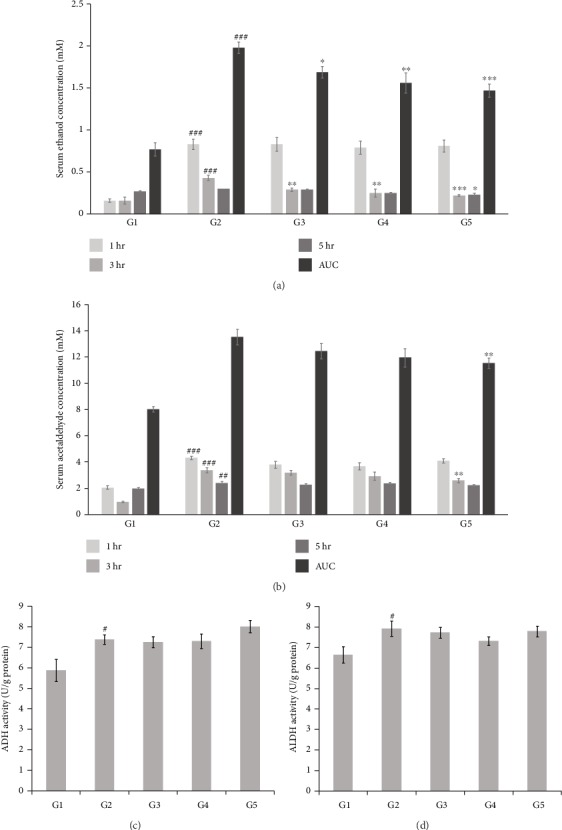
Effect of LF+AM on serum ethanol (a), acetaldehyde (b) concentration (mM) that changes due to alcohol administration and liver alcohol ADH (c), ALDH (d) activities. G1, normal control group; G2, alcohol-treated group; G3, alcohol +100 mg/kg body weight (BW) LF+AM-treated group; G4, alcohol +250 mg/kg BW LF+AM-treated group, G5; alcohol +500 mg/kg BW LF+AM-treated group. Values are expressed as mean ± SEM. #*p* < 0.05, ##*p* < 0.01, ###*p* < 0.0001 significantly different from that of G1 group ^∗^*p* < 0.05, ^∗∗^*p* < 0.01, ^∗∗∗^*p* < 0.0001 significantly different from that of G2 group. LF+AM means *Ligularia fischeri* extract and *Aronia Melanocarpa* extract complex.

The results of measuring the concentration of serum acetaldehyde, an intermediate product of alcohol metabolism, are shown in Figures 3(a) and 3(b). When only alcohol was administered (G2), serum acetaldehyde concentrations at 1, 3, and 5 h after alcohol administration showed gradual decrease over time, while 500 mg/kg LF+AM-treated group (G5) showed significant decrease at 3 h after alcohol administration. With respect to AUC, G5 showed a significantly decreasing trend. Based on the cytotoxicity test and acute model animal experiment results in this study, LF+AM could be used as safe material with similar efficacy. You et al. [26] reported on the facilitation of blood alcohol and acetaldehyde by a mixture containing *Houttuynia cordata*, and it is surmised that the material developed in the present study showed efficacy similar to this.

#### 3.4.2. Effect on Liver ADH and ALDH Activities

The results from animal experiments on the effect of LF+AM on liver ADH and ALDH activities are shown in Figures 3(c) and 3(d). The control with no alcohol administration (G1) showed ADH and ALDH activities of 5.89 ± 0.54 and 6.65 ± 0.4 U/gprotein, respectively, while the alcohol-treated group (G2) showed significant increase in ADH and ALDH activities. This indicates that alcohol metabolism system is acting normally due to alcohol consumption, and the results also showed that administration of the test article (LF+AM) had no significant effect.

In SD rats, the highest ADH and ALDH activities after alcohol consumption were found within 1 h, and relative to that point, activity showed a gradually decreasing trend as alcohol began to be metabolized [27]. Therefore, we estimated that treatment with the sample in the present study facilitated alcohol metabolism, whereby alcohol had already been completely metabolized by 3 h after consumption to reach a stable stage. So, it was difficult to investigate the effect of each samples on the liver ADH and ALDH activities in this study.

### 3.5. Animal Experiment 2: Protective Effects of LF and AM on Alcoholic Liver Damage

#### 3.5.1. Blood Ethanol Concentration

**Figure 4:**
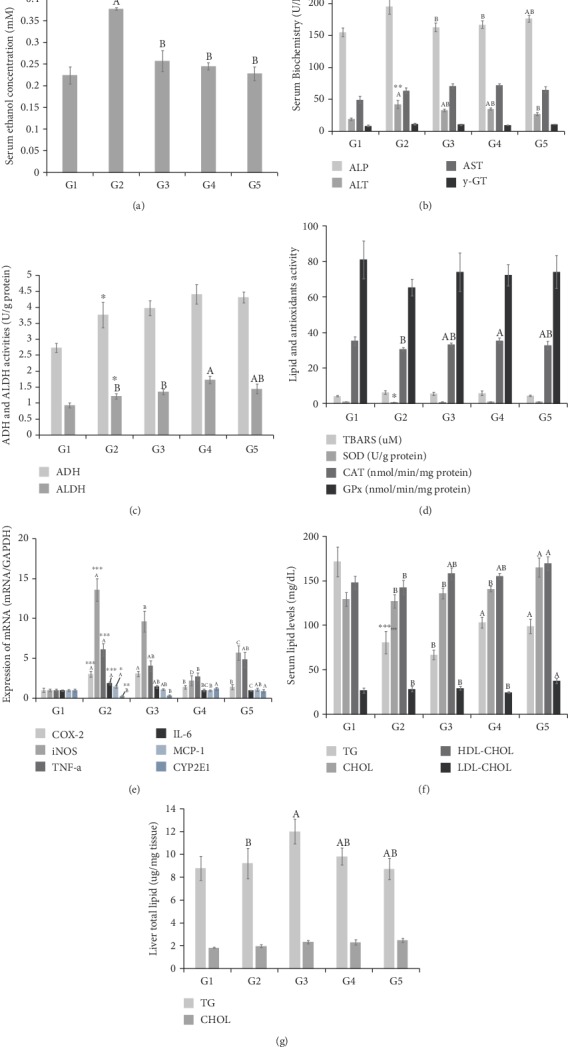
Effect of LF+AM on alcohol induced liver damage. The effect of LF+AM on serum ethanol concentration (a), serum liver injury index (b), ADH and ALDH activity (c), lipid peroxidation index (TBARS), and antioxidation activity in liver tissue (d), mRNA expression in liver tissue (e), serum lipid value (f), and liver triglyceride and cholesterol level (g). G1, normal control group; G2, alcohol-treated group; G3, alcohol +250 mg/kg body weight (BW) LF+AM-treated group; G4, alcohol +500 mg/kg BW LF+AM-treated group. Values are expressed as mean ± SEM. ^∗^*p* < 0.05, ^∗∗^*p* < 0.01, ^∗∗∗^*p* < 0.0001 significantly different from that of G2 group. Means with the different letters are significantly different (*p* < 0.05) by the Tukey's test LF+AM means *Ligularia fischeri* extract and *Aronia Melanocarpa* extract complex.

Compared with the blood ethanol concentration of the normal control group (G1), 0.22 ± 0.020 mM, the blood ethanol concentration of the alcohol control group (G2), 0.38 ± 0.003 mM, significantly increased. It was confirmed that the blood ethanol concentration significantly decreased in both of the LF+AM-treated groups (G3 and G4) with 0.26 ± 0.024 mM and 0.25 ± 0.008 mM, respectively. This result suggests that the blood ethanol metabolism system is catalyzed by orally administration of LF+AM (Figures 4(a) and 4(b).

#### 3.5.2. Liver ADH and ALDH Activities

As shown in Figure 4(c), the ADH activity of the alcohol control group (G2) was 3.77 ± 0.40 U/g protein, which has significantly increased compared with 2.74 ± 0.14 U/g protein in the normal control group (G1). The ADH activities of LF+AM-treated groups (G3 and G4) showed tendencies to increase compared with the alcohol control group (G2) but did not show any significant difference. As for ALDH activities, the alcohol control group (G2) had 1.223 ± 0.086 U/g protein, which is a significant increase compared with 0.944 ± 0.079 U/g protein in the normal control group (G1). The ALDH activity significantly increased in the high-dose LF+AM-treated group (G4) compared with that in the alcohol control group (G2). When the above blood alcohol concentration results and liver tissue ADH and ALDH activity results are compared, it is presumable that LF+AM treatment promotes alcohol metabolism by increasing the activity of ALDH.

#### 3.5.3. Effects on Hepatic Enzyme Activities

Generally, items included in a biochemistry test importantly used for liver disease diagnosis are alanine transaminase (ALT), aspartate transaminase (AST), alkaline phosphatase (ALP), *γ*-glutamyltransferase (*γ*-GT), etc. Acute or chronic liver cell damage manifests as increased blood AST or ALT. ALP, an enzyme involved in the transportation of metabolites through the cell membrane, exists on the surface of bile duct epithelial cells, and the most common pathological cause of ALP elevation is a liver disease. *γ*-GT is an enzyme distributed in the liver cells, bile duct cells, renal tubules, pancreas, intestine, etc., and similar to ALP, the mechanism for its increase in blood is liver damage.

To investigate the effects of alcohol and the test substance administration on liver functions, the activities of ALP, ALT, AST, and *γ*-GT in the serum collected at the end of the experiment were measured by using a blood biochemistry analyzer. The ALP and ALT activities in the alcohol control group (G2) significantly increased compared with the normal control group (G1). The increased ALP activity in the alcohol control group (G2) significantly decreased in the low dose and high dose LF+AM-treated groups (G3 and G4), and the AST and *γ*-GT activities did not show significant differences in all groups (Figure 4(b).

#### 3.5.4. Effects on Lipid Peroxide Formation and Antioxidant Enzyme Activity

When alcohol is continually consumed, free radicals are excessively produced as alcohol is degraded; thus, the relative function reduction of the free radical-removing antioxidant system causes imbalances and oxidative stress. The excessive oxidative stress induced damages in the cells; in particular, it continuously produces lipid peroxides by acting on the polyhydric unsaturated fatty acids of the cell membrane, thus causing cell function damage [28]. The representative antioxidant enzymes that manage the antioxidant system in the body are superoxide dismutase (SOD), catalase (CAT), glutathione peroxidase (GPx), etc. Oxygen free radicals are converted by SOD to hydrogen peroxide (H_2_O_2_), and this is again converted by CAT and GPx to water, causing detoxification. To assess the alcohol-induced oxidative stress and its effects on the antioxidant system, the levels of lipid peroxides in the liver tissue (TBARS) and the activities of the antioxidant enzymes, SOD, CAT, and GPx were measured and are shown in Figure 4(d). The TBARS level in the liver tissue was 6.31 ± 1.05 *μ*M in the alcohol-treated group (G2), which increased compared with 4.17 ± 0.41 *μ*M in the normal control group (G1) but did not show a significant difference. The TBARS levels in the liver tissue in the LF+AM-treated groups (G3 and G4) had tendencies to decrease compared with the alcohol control group (G2) but did not show significant differences. The SOD activity in the liver tissue was 0.90 ± 0.04 U/mg protein in the alcohol control group (G2), which significantly decreased compared with 1.06 ± 0.03 U/mg protein in the normal control group (G1). The SOD activity in liver tissue tended to increase due to LF+AM, but did not show significant differences. The CAT and GPx activities in the liver tissue in the alcohol control group (G2) did not show a significant difference compared with the normal control group (G1), but the CAT activity significantly increased in the high dose test substance LF+AM-treated group (G4) compared with the alcohol control group (G2).

When the above results of antioxidant enzyme activities and blood liver damage indices are combined, it appears that LF+AM treatment somewhat protects liver tissue from alcohol consumption-induced increased oxidative stress by promoting CAT activity.

#### 3.5.5. mRNA Expression in the Liver Tissue

To investigate the effects of alcohol and the test substance treatment on the expressions of inflammation-related genes (COX-2, iNOS, TNF-*α*, IL-6, and MCP-1) and alcohol metabolism-related enzyme (CYP2E1), total RNA was collected, and the real-time RT-PCR was performed. As shown in Figure 4(e), the mRNA expressions of COX-2, iNOS, TNF-*α*, IL-6, and MCP-1 dramatically increased in the alcohol control group (G2) compared with the normal control group (G1). Compared with the alcohol-treated group (G2), COX-2, TNF-*α*, MCP-1, and IL-6 mRNA expressions significantly decreased in the high dose of LF+AM-treated group (G4). Additionally, iNOS mRNA expression significantly decreased in both LF+AM-treated groups (G3 and G4). From these results, LF+AM consumption is anticipated to reduce the risk of inflammatory tissue damage in the liver, which may increase due to alcohol consumption.

Regarding the final degradation of the alcohol remaining in the body, alcohol is finally detoxified by CYP2E1, an alcohol-degrading enzyme that exists in the microsomal ethanol-oxidizing system in each cell. Compared with the normal control group (G1), CYP2E1 mRNA expression significantly decreased in the alcohol control group (G2) and significantly increased in the high dose test substance LF+AM-treated group (G4).

#### 3.5.6. Lipid Components and Contents of Liver Tissue

In long-term alcohol consumption, an initial symptom is fatty liver where triglycerides accumulate in liver cells, and it progresses to progressive damage such as fatty hepatitis, hepatitis, liver fibrosis, and liver cirrhosis. Triglyceride and total cholesterol contents in the liver tissue were measured and shown in Figure 4(f). The triglyceride content in the liver tissue did not show a difference between the normal control group (G1) and the alcohol control group (G2). The triglyceride content in the liver tissue in the low dose test substance LF+AM-treated group (G3) significantly increased compared with the alcohol control group (G2). The total cholesterol content in liver tissue did not show significant differences in all groups.

#### 3.5.7. Effects on Histomorphological Changes in Liver Tissue

**Figure 5:**
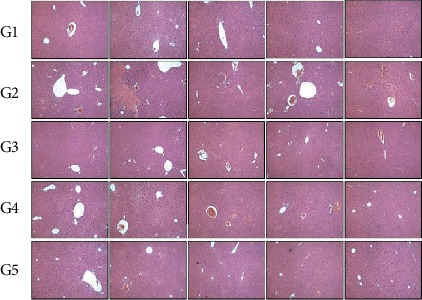
Effects on histomorphological changes in liver tissue. G1, normal control group; G2, alcohol-treated group; G3, alcohol +250 mg/kg body weight (BW) LF+AM-treated group; G4, alcohol +500 mg/kg BW LF+AM-treated group. Five liver tissues were used for each experimental groups, and the hematoxylin and eosin (H&E) stained images of live tissues were shown. LF+AM means *Ligularia fischeri* extract and *Aronia Melanocarpa* extract complex.

To investigate the histomorphological changes in the liver tissue, the results of microscopic observation after the H&E staining of the liver tissue are shown in Figure 5. No uniform liver damage or lesion was observed in the liver tissue of the normal control group (G1), but fibrosis and inflammation were observed in some parts of the liver tissue of the alcohol control group (G2). In the groups treated with the test substance LF+AM and the positive control substance (G3, G4, and G5), lymphocyte infiltration and inflammation were observed in parts of liver tissue, but these were observed to be somewhat weak lesions compared with those in the alcohol control group (G2).

## 4. Conclusions

In present study, the protective effect of LF and AM was investigated under *in vitro* and in *vivo* systems. *In vitro* experiment results showed that the activities of ADH and ALDH, which are the main enzymes on alcohol metabolism, were increased by each natural product extract and mixed composite treatment. Especially, the mixed composite (LF+AM, 9 : 1, w/w) treated cell-lines showed higher activity than each singleness extract. In the acute model animal experiment, LF+AM composite (9 : 1, w/w) treated group showed that significant decrease of blood alcohol and acetaldehyde levels than the untreated group. Considering the result of declined ALP level in G3 and G4 group of animal experiment 2, we estimated that the oral intake of LF+AM could protect liver against alcohol-induced hepatic injury.

Among the antioxidative index test, catalase activity was increased by LF+AM treatment. As well as, alcohol-induced inflammatory responses were identified to inhibit the decline of mRNA transcription promoting factors (COX-2, TNF-*α*, MCP-1, and IL-6). Therefore, the protection effect of LF+AM composite against alcoholic liver damage is because of involving on the hepatic antioxidation and anti-inflammatory effect.

These results showed a similar pattern to other existing studies showing the protective effect of alcoholic liver disease of CQAs, which were selected as an indicator component of LF [29] and are presumed to exhibit a synergistic effect with C3G, which is the indicator component of AM. Meanwhile, several studies have been conducted to overcome the low bioavailability of C3G, which is an indicator of AM, and through these studies, it was known that C3G is partially absorbed in not only small intestine but also in the gastric wall and colon [30]. However, further studies should be conducted to figure out the direct correlation of C3G and protective effect against alcoholic liver. As well as, the stability of C3G or CQAs during distribution and preservation should be investigated.

## Figures and Tables

**Table 1:** RT-PCR primer information.

mRNA	Primer sequences	
	Forward	Reverse
COX-2	5′-AACCGCATTGCCTCTGAAT-3′	5′-CATGTTCCAGGAGGATGGAG-3′
iNOS	5′-CGAAACGCTTCACTTCCAA-3′	5′-TGAGCCTATATTGCTGTGGCT-3′
TNF-*α*	5′-ATGAGCACAGAAAGCATGA-3′	5′-AGTAGACAGAAGAGCGTGGT-3′
IL-6	5′-CCTCTGGTCTTCTGGAGTACC-3′	5′-ACTCCTTCTGTGACTCCAGC-3′
MCP-1	5′-TCCCACTCACCTGCTGCTACTCA-3′	5′-GCTTCTTTGGGACACCTGCTG-3′
CYP2E1	5′-GTTGCCTTGCTTGTCTGGAT-3′	5′-AGGAATTGGGAAAGGTCCTG-3′
GAPDH	5′-TGGGTGTGAACCATGAGAAG-3′	5′-GCTAAGCAGTTGGTGGTGC-3′

## Data Availability

The data used to support the findings of this study are available from the corresponding author upon request.
